# Prognostic performance of early immune and endothelial activation markers in mild-to-moderate COVID-19 outpatients: a nested case-control study

**DOI:** 10.3389/fimmu.2024.1501872

**Published:** 2024-11-27

**Authors:** Andrea Alemany, Núria Balanza, Pere Millat-Martinez, Dan Ouchi, Marc Corbacho-Monné, Cristian Morales-Indiano, Gema Fernández Rivas, Ignacio Blanco, Oriol Mitjà, Ruth Aguilar, Carlota Dobaño, Quique Bassat, Gemma Moncunill, Bàrbara Baro, COnV-ert BMK STUDY GROUP

**Affiliations:** ^1^ ISGlobal, Barcelona, Spain; ^2^ Fight Infectious Diseases Foundation, Badalona, Spain; ^3^ Hospital Universitari Germans Trias i Pujol, Badalona, Spain; ^4^ Facultat de Medicina i Ciències de la Salut, Universitat de Barcelona (UB), Barcelona, Spain; ^5^ Hospital Universitari Parc Taulí, Sabadell, Spain; ^6^ Clinical Laboratory Metropolitana Nord, Badalona, Spain; ^7^ Microbiology Department, Clinical Laboratory Metropolitana Nord, Barcelona, Spain; ^8^ CIBER Enfermedades Infecciosas (CIBERINFEC), Instituto de Salud Carlos III, Madrid, Spain; ^9^ CIBER de Epidemiología y Salud Pública, Instituto de Salud Carlos III, Madrid, Spain; ^10^ Consorcio de Investigación Biomédica en Red de Epidemiología y Salud Pública (CIBERESP), Madrid, Spain; ^11^ ICREA, Pg Lluís Companys 23, Barcelona, Spain; ^12^ Pediatrics Department, Hospital Sant Joan de Déu, Universitat de Barcelona, Esplugues, Barcelona, Spain; ^13^ Centro de Investigação em Saúde de Manhiça (CISM), Maputo, Mozambique

**Keywords:** biomarkers, host response, COVID-19, SARS-CoV-2, outpatients

## Abstract

**Introduction:**

Evidence on the association of biomarkers of host response to infection with COVID-19 clinical outcomes has focused mainly on hospitalized patients. We investigated the prognostic performance of 39 immune and endothelial activation markers measured early in the course of disease to predict the development of severe COVID-19 and hospitalization.

**Methods:**

We conducted a nested case-control study from a randomized clinical trial evaluating the efficacy of COVID-19 convalescent plasma in outpatients aged 50 years or older presenting with mild-to-moderate COVID-19. We selected participants who were hospitalized within 28 days (cases) and who were not (controls) to compare their biomarker levels in plasma samples collected at enrolment.

**Results:**

A total of 42 cases and 42 controls were included in this study. The levels of CRP, IL6, IP10, ferritin, IFNα, IL8, IL1RA, MCP1, and RANTES, determined within 7 days of symptoms onset, showed good individual prognostic performance for COVID-19 associated hospitalization by day 28. The biomarkers CRP, IL6, IP10, IL8, IL1RA, and suPAR showed good individual prognostic performance for severe COVID-19. CRP, IL6 and IP10 had the most robust association with both hospitalization and severe COVID-19, with CRP having the highest discriminatory capacity with hospitalization, and IL6 for severe COVID-19.

**Discussion:**

Our study shows good prognostic performance of CRP and IL6 for 28-day hospitalization in patients with mild-to-moderate COVID-19, in the absence of clinical criteria for admission upon enrolment. These findings confirm the value of these biomarkers at early stages of COVID-19 disease in the outpatient setting to support management decisions.

## Introduction

COVID-19 can rapidly progress to severe disease, leading to acute respiratory distress, multi-organ failure, and death, particularly in individuals with underlying conditions, weakened immune systems, and unvaccinated (1). COVID-19 symptoms and risk factors have shown poor specificity and thus limited accuracy for early identification of patients at risk (1–3), hence the role of host biomarkers in predicting severe outcomes has been investigated to enhance triaging strategies. Several biomarkers of inflammation, thrombosis, blood count and coagulation, end-organ damage, as well as immune and endothelial activation have shown to be associated with oxygen requirement, non-invasive ventilation, intensive care admission, and in-hospital mortality.

Most of the evidence has focused on hospitalized patients at a severe stage of the disease. Meta-analyses compiling the initial evidence, mainly based on retrospective studies involving hospitalized patients in China, highlighted the association of disease severity and mortality with lymphopenia, thrombocytopenia, elevated levels of C-reactive protein (CRP), procalcitonin (PCT), lactate dehydrogenase (LDH), D-dimer, ferritin, interleukin 6 (IL6), E-selectin, vascular cell adhesion molecule 1 (VCAM1), and Angiopoietin 2 (Angpt2), among others (4–8). These meta-analyses showed high heterogeneity between studies in severity outcomes, which hindered interpretation of biomarkers’ utility. Prospective studies of hospitalized patients confirmed these associations, also for markers related to immune activation such as interleukin 8 (IL8), soluble urokinase plasminogen activator receptor (suPAR), and soluble triggering receptor expressed on myeloid cells 1 (sTREM1) (9–12). Of note, a large prospective cohort of 1,484 admitted patients showed strong association with disease severity and death of elevated levels of IL6 and tumour necrosis factor (TNF) on admission (13). In a smaller prospective cohort of 187 inpatients, IL6 and suPAR showed the best area under the receiver operating characteristic curve (AUROC) for predicting severe COVID-19 (14), although their performance was nearly comparable to that of age and the National Early Warning Score 2.

Evidence on early biomarkers assessment prior to the onset of severe COVID-19 and hospitalization is rather scarce. A prospective study of 76 patients presenting to an emergency department with mild, moderate, or severe COVID-19 showed strong prognostic accuracy for sTREM1 and IL6, with sTREM1 having the best AUROC for intubation/mortality (0.86; 95% CI: 0.77-0.95) and IL6 for oxygen requirement (0.84; 95% CI: 0.74-0.94) (15). Another prospective study of 426 patients with moderate COVID-19 presenting to two hospitals in India developed and validated three clinical prediction models (particularly useful for resource-limited settings) using age, sex, SpO2, and either neutrophil to lymphocyte ratio, suPAR, or IL6 levels to identify patients unlikely to need supplemental oxygen and hence suitable for safely discharge and community management (16). Hence, it remains to be further demonstrated the prognostic value of these promising biomarkers in early mild COVID-19, to confirm their potential to guide triage and management decisions.

The main aim of this study was to investigate the prognostic performance of 39 immune and endothelial activation markers, measured in patients with mild-to-moderate COVID-19 within 7 days of symptoms onset and discharged home after first clinical assessment, to predict progression to hospitalization and severe COVID-19.

## Methods

### Study design

We conducted an unmatched nested case-control study from a multicenter, double-blind, randomized, placebo-controlled trial assessing the efficacy of COVID-19 convalescent plasma in preventing severe COVID-19 and hospitalization up to day 28 in patients infected with SARS-CoV-2 with mild-to-moderate illness (COnV-ert trial, NCT04621123) (17). Recruitment was conducted between November 2020 and July 2021 in four health-care centers in Spain.

The COnV-ert trial and the blood sample analysis were approved by the Ethics Committee at Hospital Germans Trials i Pujol (number PI 20-313) and the institutional review boards of all participating centers. All participants provided written informed consent before enrolling in the study.

### Study population

Eligible participants for the COnV-ert study were aged 50 years or older, regardless of other risk factors for severe disease, and with confirmed SARS-CoV-2 infection within 5 days from enrolment. All patients were non-hospitalized and with mild-to-moderate COVID-19 upon first presentation, with symptom onset within the previous 7 days. A total of 376 participants were enrolled in the COnV-ert study; and hospitalization up to 28 days occurred in 43/376 participants (11.4%), with no significant differences between the convalescent plasma and the placebo groups.

For this study, we selected participants from the COnV-ert study based on hospitalization status up to day 28 for additional biomarker investigation in plasma samples collected at enrolment and day 7. We included all cases who required hospitalization for COVID-19 progression within 28 days after enrolment and who had available baseline plasma sample. For the control group, we randomly selected participants who did not require hospitalization for COVID-19 up to 28 days at a 1:1 ratio.

### Clinical and laboratory evaluation

As part of the COnV-ert study procedures, baseline demographic and clinical characteristics, including risk factors and COVID-19 symptoms and severity, were collected using standardized questionnaires and recorded using a structured electronic case report form. Follow-up visits were performed on days 3, 7, 14, 28, and 60, to assess hospitalization status and disease severity. Severity of COVID-19 was assessed using the WHO Clinical Progression Scale, a validated score that ranges from 0 (not infected), 1-3 (ambulatory mild disease), 4-5 (hospitalized with moderate disease), 6-9 (hospitalized with severe disease) and 10 (dead) (18). Blood samples for biomarker assessment were collected at enrolment, before administration of interventional product, and 7 days after enrolment and administration of interventional product, using EDTA coated tubes.

Blood levels of the inflammatory biomarkers D-dimer, ferritin, IL6, lymphocytes, CRP, and pre-albumin for all participants included were quantified on the same day at the centralized clinical laboratory of *Hospital Universitari Germans Trias i Pujol.* Plasma D-dimer concentration was measured using a latex-enhanced immunoassay (ACL TOP 750 analyzer, Instrumentation Laboratory), IL6 levels by electrochemiluminiscent immunoassay of paramagnetic particles (Unicel DxI 800 analyzer, Beckman Coulter), CRP, ferritin and pre-albumin were determined by immunoturbidimetry assay (AU5800 analyzer, Beckman Coulter), and lymphocytes count was measured by VCSn technology (DxH900 analyzer, Beckman Coulter). Procedures were performed according to manufacturers’ instructions and blinded to clinical data.

Additional biomarker quantification was retrospectively performed at a centralized laboratory in ISGlobal for the participants included in this study, on samples stored at -80°C without freeze-thaw until batch analyte quantification. The concentrations of a panel of 30 cytokines, chemokines and growth factors were measured in plasma samples by Luminex using the Cytokine Human Magnetic 30-Plex Panel LHC6003M from Life Technologies™. This panel included the following analytes which are involved in inflammatory or immune responses: epidermal growth factor (EGF), eotaxin, fibroblast growth factor (FGF), granulocyte colony-stimulating factor (G-CSF), granulocyte-macrophage colony-stimulating factor (GM-CSF), hepatocyte growth factor (HGF), interferon-alpha (IFNα), IFNγ, interleukin-1 receptor agonist (IL1RA), IL1β, IL2, IL2R, IL4, IL5, IL6, IL7, IL8, IL10, IL12 (p40/p70), IL13, IL15, IL17, IFNγ induced protein 10 (IP10), monocyte chemoattractant protein 1 (MCP1), monokine induced by IFNγ (MIG), macrophage inflammatory protein 1α (MIP1α), MIP1β, Regulated upon Activation, Normal T Cell Expressed and Presumably Secreted (RANTES), Tumor necrosis factor (TNF), and vascular endothelial growth factor (VEGF). Briefly, 25 μL of plasma were tested, applying a modification to the manufacturer’s protocol by using half the volume of all reagents including the standards. This modification was previously tested and showed no difference in assay performance compared to the original protocol and has been used in prior studies (19–21). Each plate included 16 serial dilutions (2-fold) of a standard sample with known concentrations of each analyte and two blanks. In addition, concentrations of Angpt2, IL8, PCT, sTREM1, and suPAR were also measured in plasma samples by Luminex using a custom-developed panel from R&D Systems (LXSAHM) following manufacturer instructions. Each plate included 11 serial dilutions (2-fold) of a standard sample with known concentrations of each analyte and two blanks. For both kits, samples were acquired on a Luminex^®^100/200™ instrument and analyzed with xPONENT^®^ software 3.1. The concentration of each analyte was obtained by interpolating the median fluorescent intensity (MFI) to a 5-parameter logistic regression curve and reported as pg/mL using the drLumi R package. Limits of quantification (LOQ) were estimated based on cut-off values of the 30% coefficient of variation (CV) of the standard curve for each analyte. When the value of an analyte was below the lower LOQ (LLOQ), a random value between the LLOQ and the LLOQ/2 was assigned. One biomarker from the 30-plex panel (interleukin 15) was excluded from the analyses as more than 30% of the values were under the LLOQ.

### Study endpoints

The primary endpoint was hospitalization due to COVID-19 progression within 28 days of enrolment (WHO Clinical Progression scale score 4 to 10). Secondary endpoints included (1) hospitalization with severe COVID-19, defined as hospitalization with requirement of oxygen by non-invasive ventilation or high flow, intubation and mechanical ventilation, or death (WHO Clinical Progression scale score 6 to 10) within 28 days of enrolment, and (2) hospitalization with moderate disease, either with no need for oxygen therapy or with oxygen by mask or nasal prongs (WHO Clinical Progression scale score 4 to 5).

The decision to hospitalize was based on clinical judgement and national guidelines, which were standardized across all study sites, without the use of biomarker information. The secondary endpoint of severe COVID-19, presented with lower frequency, was added as it provides standard definition of severity and allows greater generalizability across different settings.

### Statistical methods

All analyses were performed in all study participants with complete baseline biomarker information for all analytes of interest, defining the analysis population. Descriptive data were summarized using medians [interquartile range, IQR] or counts (%).

To investigate the association of the concentration of biomarkers with primary and secondary endpoints, we performed several analyses. Biomarker data were log-transformed for inclusion in all regression models. First, we selected the best discriminatory biomarkers for further exploration using nonparametric statistical methods to compare levels of 39 biomarkers at baseline with hospitalization and severe COVID-19 up to day 28. The Kruskal-Wallis test and *post-hoc* comparisons with Dunn’s test were used to assess differences in biomarker levels between the three groups: i) non-hospitalized, ii) hospitalized with moderate disease (WHO Clinical Progression scale score 4 to 5), and iii) hospitalized with severe disease (WHO Clinical Progression scale score 6 to 10), with p-values adjusted for multiple comparisons using Holm’s method. We presented a heatmap with the median levels of the biomarkers for each group, and a dendrogram clustering the biomarkers using Ward’s D2 method with Euclidean distances. Second, logistic models were used to estimate odds ratios (ORs) for hospitalization and severe COVID-19 of the selected biomarkers. For logistic models, we estimated univariable and multivariable models accounting for relevant covariates. Third, the discrimination capacity of each biomarker was assessed using AUROCs, which were analyzed nonparametrically and compared pairwise using the algorithm suggested by DeLong et al. (22). Fourth, for the best biomarkers, performance metrics associated with different cut-off levels were calculated. We used cut-offs described in the literature and derived the best ones for our cohort according to the maximum Youden index.

As a secondary aim, we assessed levels of biomarkers at baseline and day 7. Baseline biomarkers levels according to study endpoints were compared non-parametrically using Kruskal-Wallis tests, followed by Dunn’s tests with Holm correction in three groups. In a subset of individuals with biomarker data at day 7, biomarkers kinetics were explored by determining mean changes from baseline to day 7. The mean change of each biomarker was compared, by severity or treatment groups, through fitting linear mixed-effects models, using the individual as random effects in the intercept to adjust for intra-individual correlation and modeling time-group interaction effects. Last, correlation of biomarkers level with viral load was assessed using Spearman’s rank correlation coefficients.

All analyses were performed using Stata v16.1 (Stata Corp, College Station, TX, USA) and R statistical package version 4.3 or higher statistical packages, under a two-tailed significance level of 0.05.

Our unmatched case-control study with a total sample size of 79 participants with a 1:1 ratio (38 cases and 41 controls) has 80% statistical power to detect a minimum relative increase of 44% in the mean levels of CRP marker on log2 scale in the cases vs control group, using a two-sided significance level of 0.05.

## Results

### Study population characteristics

A total of 84 COnV-ert participants with available baseline plasma samples were included in this study; 42 hospitalized participants (cases) and 42 non-hospitalized (controls). 79/84 participants (38/42 cases and 41/42 controls) had complete baseline biomarker information for all 39 analytes of interest and were included in all analyses (Study flowchart – **Supplementary Figure S1**).

Baseline demographic and clinical characteristics were similar between cases and controls (**Table 1**). The median age was 58 (IQR 54 to 66) years, 37/79 (47%) were female, and obesity (39%) was the most frequent coexisting comorbidity. Viral load was significantly higher in cases than controls. The median time from symptom onset to enrolment and sample collection was 5 days (IQR 3 to 6). 42/79 (53%) participants were randomized to receive convalescent plasma infusion. Within the cohort of hospitalized participants, 13/38 (34%) fulfilled the criteria of severe COVID-19 disease according to the WHO Clinical Progression Scale (scores 6 to 10), 5/38 (13%) individuals required mechanical ventilation, and 2/38(5%) died. Median time from recruitment and sample collection to hospitalization was 4 days (IQR 3 to 5).

**Table 1:** Analysis population characteristics.

Characteristics (n=79) N (%) or median [IQR]^a^	Total (n=79)	Hospitalized (cases) (n=38)	Non-hospitalized (controls) (n=41)	p-value^b^
Demographics				
Male sex	42 (53.2)	20 (52.6)	22 (53.7)	0.927
Female sex	37 (46.8)	18 (47.4)	19 (46.3)	0.927
Age (years)	58 [54, 66]	60 [54, 68]	57 [53, 61]	0.242
BMI (kg/m^2^)	29.1 [26.8, 32.5]	29.5 [26.9, 32.8]	28.8 [26.6, 31.3]	0.530
Smoker	43 (54.4)	17 (44.7)	26 (63.4)	0.096
COVID-19 infection at baseline				
Days from symptoms onset to recruitment	5 [3, 6]	5 [4, 6]	4 [3, 6]	0.544
Days from positive test to recruitment	3 [2, 4]	3 [2, 4]	3 [2, 3]	0.401
IgM and IgG negative status	69 (87.3)	34 (89.5)	35 (86.4)	0.583
Viral load (log_10_ copies/mL)^c^	6.8 [5.9, 7.6]	7.1 [6.5, 7.9]	6.2 [4.9, 7.3]	0.001
Coexisting comorbidities				
Obesity (BMI>30)	31 (39.2)	17 (44.7)	14 (34.2)	0.335
Cardiovascular disease	8 (10.1)	4 (10.5)	4 (9.8)	0.910
Lung disease (COPD, asthma, or both)	7 (8.9)	4 (10.5)	3 (7.3)	0.616
Diabetes	8 (10.1)	2 (5.3)	6 (14.6)	0.168
Cancer	4 (5.1)	2 (5.3)	2 (4.9)	0.938
Immune-compromised	3 (3.8)	1 (2.6)	2 (4.9)	0.602
Neurological disease	2 (2.5)	1 (2.6)	1 (2.4)	0.957
Liver disease	3 (3.8)	0 (0)	3 (7.3)	0.089
Intervention group				
Placebo	37 (46.8)	17 (44.7)	20 (48.8)	0.719
CCP	42 (53.2)	21 (55.3)	21 (51.2)	
Evolution of disease: Maximum WHO COVID-19 severity scale by day 28:				
Score 2 to 3 – ambulatory mild disease	41 (51.9)	0 (0)	41 (100)	<0.001
Score 4 to 5 – hospitalized with moderate disease	25 (31.7)	25 (65.8)	0 (0)	
Score 6 to 9 – hospitalized with severe disease	11 (13.9)	11 (29.0)	0 (0)	
Score 10 – hospitalized and death	2 (2.5)	2 (5.3)	0 (0)	

^a^Data presented as frequency (percent) or median [interquartile range] as appropriate.

^b^p-values computed using Chi-squared or Mann-Whitney U tests.

^c^Missing data: viral load = 1.

### Selection of best discriminatory biomarkers for further exploration

**Figure 1:**
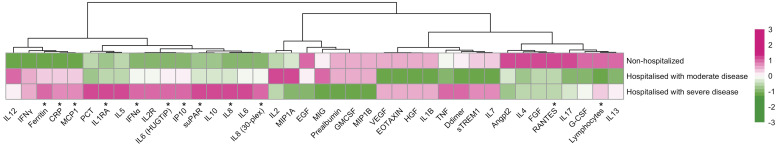
Heatmap: selection of best discriminatory biomarkers. Legend: Heatmap of median levels of the 39 biomarkers of interest with severity of disease achieved within 28 days: (i) non-hospitalized, (ii) hospitalized with moderate COVID-19 disease (WHO Clinical Progression Scale score 4 to 5), (iii) hospitalized with severe COVID-19 disease (WHO Clinical Progression Scale score 6 to 10). P-values were calculated using Krustal-Wallis test. Statistically significant differences are indicated with asterisk (*).

Of the 39 biomarkers of interest, 11 biomarkers showed an overall significant difference in median baseline biomarker levels based on hospitalization and COVID-19 severity up to day 28, including CRP, ferritin, IFNα, IL6, IL8, IL1RA, IP10, MCP1, RANTES, suPAR, and lymphocytes (**Figure 1**). Lymphocytes were excluded as levels were not changing sequentially with severity, and the remaining 10 biomarkers were selected for further exploration. Prognostic markers previously highlighted in the literature such as Angpt2, sTREM1, and PCT didn’t show significant differences overall when considering these three groups. Interestingly, our findings showed that while hospitalized participants had lower lymphocyte levels compared to controls (median 1.2x10^9^ cells/L, IQR 1.0 to 1.6), those hospitalized with moderate disease exhibited lower lymphocyte levels than those hospitalized with severe disease (median 0.9x10^9^ cells/L, IQR 0.8 to 1.2 vs median 1.1x10^9^ cells/L, IQR 0.8 to 1.2).

### Biomarkers as indicators of hospitalization and severe COVID-19

We observed a significant increased odds of hospitalization within 28 days from enrolment for every two-fold increased level of the biomarkers CRP, IL6, IP10, ferritin, IFNα, IL8, IL1RA, MCP1, and a decrease of RANTES (**Table 2**). Results were similar after adjustment for age, sex, and BMI. When adjusting for viral load, IFNα, IL8, IL1RA, and MCP1 lost significance, whereas suPAR gained significance. We also observed a significant increased odds of severe COVID-19 (WHO Clinical Progression Scale score 6 to 10) for increased concentration of CRP, IL6, IP10, IL8, IL1RA, and suPAR (**Table 2**). After adjustment for age, sex, and BMI, ferritin showed a significant increase, suPAR lost significance, and the rest of biomarkers’ associations remained unchanged. After adjusting for viral load, IL1RA was no longer significant. Adjustment for viral load was conducted as baseline viral load showed an independent association with both hospitalization and severe COVID-19, with an odds ratio of 1.71 for every ten-fold increase (95% CI 1.20 to 2.45; p=0.03 for both outcomes).

**Table 2:** Associations between biomarker levels and endpoints in the analysis population.

Primary endpoint: Hospitalization within 28 days after enrolment						
Biomarker	Unadjusted OR^a^	p-value	Adjusted OR^b^	p-value	Adjusted OR^c^	p-value
CRP	2.39 (1.60, 3.58)	<0.001	2.37 (1.58, 3.55)	<0.001	2.96 (1.74, 5.03)	<0.001
Ferritin	2.16 (1.36, 3.42)	0.001	2.96 (1.64, 5.35)	<0.001	3.23 (1.74, 6.01)	<0.001
IFNα	1.53 (1.11, 2.11)	0.010	1.56 (1.11, 2.18)	0.010	1.38 (0.98, 1.94)	0.063
IL6	3.23 (1.87, 5.57)	<0.001	3.25 (1.87, 5.65)	<0.001	4.99 (2.25, 11.04)	<0.001
IL8	1.78 (1.10, 2.87)	0.019	1.81 (1.12, 2.94)	0.016	1.53 (0.93, 2.53)	0.095
IL1RA	1.73 (1.05, 2.82)	0.030	1.79 (1.07, 3.00)	0.027	1.57 (0.92, 2.67)	0.096
IP10	3.01 (1.61, 5.64)	0.001	2.95 (1.57, 5.56)	0.001	2.52 (1.30, 4.87)	0.006
MCP1	1.88 (1.06, 3.33)	0.030	1.88 (1.05, 3.36)	0.033	1.59 (0.88, 2.89)	0.124
RANTES	0.15 (0.04, 0.61)	0.008	0.14 (0.03, 0.57)	0.006	0.23 (0.06, 0.97)	0.046
suPAR	1.67 (0.99, 2.81)	0.053	1.69 (0.98, 2.90)	0.057	1.89 (1.04, 3.41)	0.036
Secondary endpoint: Severe COVID-19* within 28 days after enrolment						
Biomarker	Unadjusted OR^a^	p-value	Adjusted OR^b^	p-value	Adjusted OR^c^	p-value
CRP	1.81 (1.20, 2.71)	0.004	1.81 (1.18, 2.78)	0.007	2.12 (1.28, 3.50)	0.003
Ferritin	1.71 (1.00, 2.93)	0.052	2.50 (1.17, 5.36)	0.018	2.08 (1.08, 3.99)	0.028
IFNα	1.37 (0.90, 2.11)	0.146	1.47 (0.91, 2.36)	0.113	1.25 (0.80, 1.96)	0.335
IL6	2.39 (1.36, 4.19)	0.002	2.43 (1.32, 4.46)	0.004	3.00 (1.46, 6.15)	0.003
IL8	2.48 (1.23, 5.02)	0.011	2.72 (1.28, 5.77)	0.009	2.29 (1.11, 4.73)	0.025
IL1RA	2.14 (1.04, 4.39)	0.038	2.84 (1.21, 6.66)	0.016	1.98 (0.95, 4.11)	0.067
IP10	3.42 (1.35, 8.63)	0.009	3.52 (1.28, 9.66)	0.015	3.18 (1.18, 8.61)	0.023
MCP1	1.53 (0.79, 2.96)	0.203	1.60 (0.79, 3.23)	0.192	1.30 (0.66, 2.58)	0.443
RANTES	0.58 (0.15, 2.31)	0.442	0.48 (0.11, 2.23)	0.353	0.86 (0.17, 4.41)	0.858
suPAR	2.50 (1.07, 5.84)	0.034	2.38 (0.99, 5.73)	0.053	2.87 (1.14, 7.27)	0.026

^*^Severe COVID-19 was defined as a score 6 to 10 in the WHO Clinical Progression Score scale.

^a^ORs, 95% CI and p-values are from univariable logistic regression models.

^b^ORs, 95% CI and p-values are from multivariable logistic regression models adjusting for age, sex, and BMI.

^c^ORs, 95% CI and p-values are from multivariable logistic regression models adjusting for baseline viral load.

All ORs presented indicate the increase in the odds of the outcome for every two-fold increase in each biomarker concentration.

**Figure 2:**
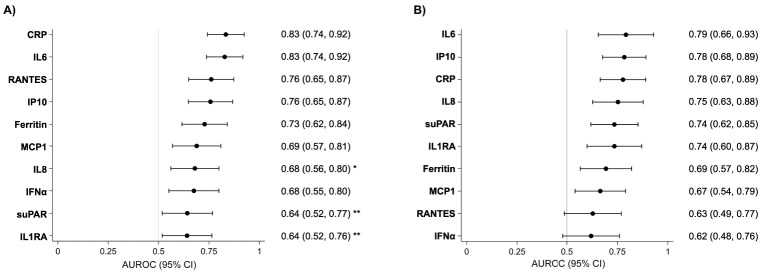
AUROC of each biomarker for hospitalization and severe COVID-19 within 28 days. Legend: Forest plot showing AUROC for hospitalization **(A)** and for severe COVID-19 **(B)** within 28 days after enrolment of the 10 selected biomarkers. Dots show AUROC with lines indicating 95% CI. Differences in AUROC between the top-ranking biomarker and other biomarkers were tested using methods recommended by DeLong et al., with an asterisk (*) indicating the AUROC is significantly different to the best one (*p*<*0.05, **<0.01, ***p*<*0.001).

The biomarkers with the best discrimination capacity for hospitalization were CRP and IL6 (AUROC of 0.83 95% CI: 0.74 to 0.92; and 0.83 95% CI: 0.74 to 0.92, respectively), followed by RANTES, IP10, ferritin, MCP1, IFNα (AUROCs of 0.76 95% CI 0.65 to 0.87; 0.76 95% CI: 0.65 to 0.87; 0.73 95% CI: 0.62 to 0.84; 0.69 95% CI: 0.57 to 0.81; and 0.68 95% CI 0.55 to 0.80), which were not significantly different predictors (**Figure 2A**). The biomarker with the best discrimination capacity for severe COVID-19 was IL6, with an AUROC of 0.79 (95%CI: 0.66 to 0.93), closely followed by IP10 and CRP (AUROCs of 0.78, 95% CI 0.68 to 0.89; and 0.78, 95% CI 0.67 to 0.89). However, these three markers did not show higher discriminatory capacity for severity compared to IL8, suPAR, IL1RA, ferritin, MCP1, IFNα, and RANTES (**Figure 2B**).

We calculated the sensitivity and specificity of CRP and IL6, using the established laboratory reference ranges or cut-off values, to predict hospitalization and severe COVID-19, respectively, in our study cohort (**Supplementary Table S1**). We obtained high sensitivities of 90% and 92%, respectively. However, while specificity was relatively high for CRP with hospitalization (63%), IL6 showed low specificity to predict severity (44%). We also explored the cut-off values within our cohort that maximize sensitivity and specificity (**Supplementary Table S1**).

Finally, viral load showed no significant correlation with the selected biomarkers, except for IFNα, IP10, MCP1 and RANTES, with IP10 demonstrating the strongest correlation (**Supplementary Figure S2**).

### Biomarker levels and kinetics by day 7

**Figure 3:**
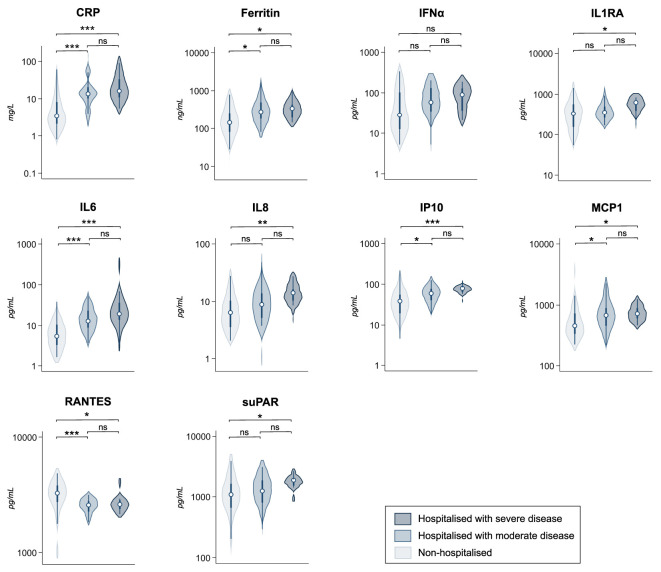
Distribution of levels of immune and endothelial activation biomarkers according to hospitalization status and COVID-19 severity within 28 days. Legend: Violin plots showing the distribution of levels of 10 biomarkers of immune and endothelial activation according to hospitalization status and COVID-19 severity within 28 days. Dots indicate median values, with lines indicating IQR. Groups: 0=Non hospitalized (controls), 1=Hospitalized with moderate disease (WHO Clinical Progression Scale score 4 to 5), 2=Hospitalized with severe disease (WHO Clinical Progression Scale score 6 to 10). P-values were calculated using Pairwise Dunn’s test and adjusted by Holm method. Statistically significant differences are indicated with asterisk (*p<0.05, **<0.01, ***p<0.001, ns, non significant).

Distribution of biomarker levels at baseline according to hospitalization status and COVID-19 severity within 28 days is shown in **Figure 3**. Participants hospitalized with moderate disease (WHO Clinical Progression Scale score 4 to 5), compared to controls, showed significantly higher baseline median levels of CRP, IL6, IP10, ferritin, and MCP1; but not IL8, IL1RA, IFNα, and suPAR, and significantly lower levels of RANTES. Participants hospitalized with severe COVID-19 (WHO Clinical Progression Scale score 6 to 10), showed significantly higher baseline median levels of all selected biomarkers, except for IFNα, and significantly lower levels of RANTES, compared to controls.

**Figure 4:**
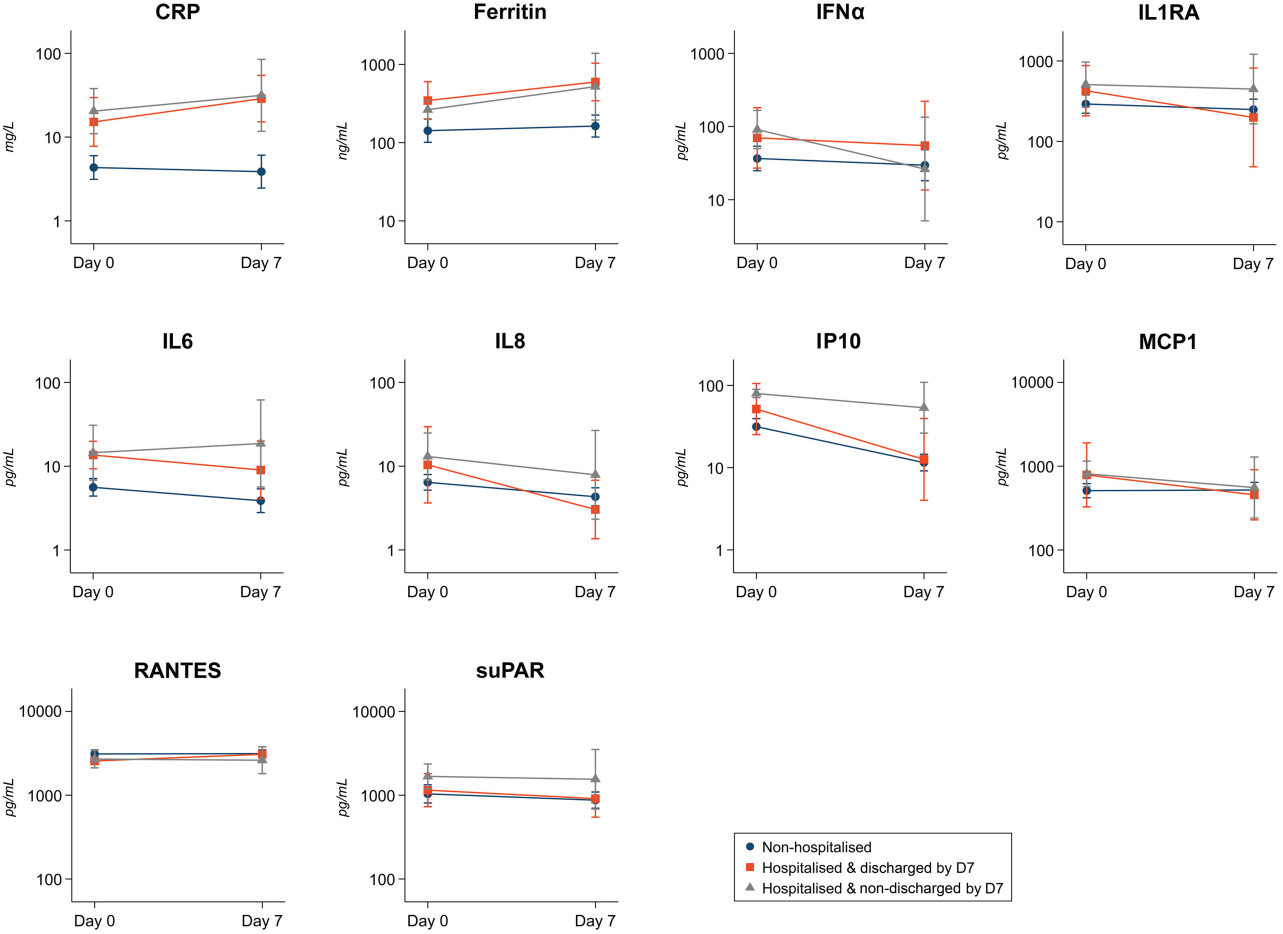
Biomarkers kinetics between baseline and day 7. Legend: Figure shows the kinetics of the 10 selected biomarkers from baseline to day 7 after enrolment in a subset of individuals with available biomarker data on day 7 in three severity groups: (i) non-hospitalized up to day 28 (controls) in blue color; (ii) hospitalized but already discharged at day 7 (cases with shorter hospitalization) in orange; (iii) still hospitalized at day 7 (cases with longer hospitalization) in grey. Figure shows mean (dots) in log_10_ biomarker levels and 95% CI (vertical lines), and lines connect mean levels between baseline (day 0) and day 7. The mean difference of each biomarker level and the number of participants with available biomarker data on day 7 for each severity group is shown in **Supplementary Table S1**.

Biomarker kinetics were explored by determining the mean change in levels of each biomarker (in log_10_ scale) from baseline to day 7 after enrolment and administration of investigational product. This was done only in a subset of individuals with available sample on day 7 for biomarker’s assessment. We compared biomarkers dynamics between three groups, according to hospitalization status and length of hospitalization as a proxy of severity/recovery: (1) patients non-hospitalized up to day 28 (controls), (2) patients hospitalized but already discharged at day 7 (cases with shorter hospitalization), and (3) patients still hospitalized at day 7 (cases with longer hospitalization) (**Figure 4**; **Supplementary Table S2**). Most biomarkers’ levels showed a decrease between baseline and day 7 in non-hospitalized participants, in line with lack of progress towards severity. However, a trend in increasing levels of CRP and ferritin was observed in participants who required hospitalization within 28 days, regardless of hospitalization status at day 7, which showed statistical significance only for ferritin. Moreover, a trend in higher mean levels of IL6, IP10, IL8, IL1RA, and suPAR was observed only in participants still hospitalized at day 7, compared to those already discharged at day 7 and controls. We did not observe significant differences in kinetics for any of the selected biomarkers according to the interventional treatment received at baseline (convalescent plasma or placebo), except for IL1RA (**Supplementary Table S3**).

## Discussion

In this nested case-control study in mild-to-moderate COVID-19 outpatients, the concentration of the biomarkers CRP, IL6, IP10, ferritin, IFNα, IL8, IL1RA, MCP1, and RANTES determined within 7 days of symptoms onset showed good individual prognostic performance for progression towards hospitalization due to COVID-19 within 28 days. The biomarkers CRP, IL6, IP10, IL8, IL1RA, and suPAR showed also a good performance in identifying those participants at risk of developing severe COVID-19, defined as patients who required oxygen by non-invasive ventilation or high flow, intubation and mechanical ventilation, or who died. These results are in line with large body of evidence showing that several immune activation and inflammatory biomarkers are associated with progression to severe COVID-19. Importantly, we confirmed their prognostic potential when assessed early in the course of disease, prior to the development of clinically noticeable severe manifestations and hospitalization requirement.

Interestingly, CRP, IL6 and IP10 had the most robust association with hospitalization and severe COVID-19, with CRP having the highest discriminatory capacity for hospitalization, and IL6 for severe COVID-19. These results confirm the early prognostic capacity of IL6 to predict oxygen requirement, as observed in two prospective studies of patients presenting with COVID-19 to emergency services (15, 16), given that severity in our study participants was primarily associated with oxygen needs rather than mortality. Unlike our study, these prospective studies found limited prognostic value of CRP levels, especially to discriminate hospital requirement. Although CRP has been largely associated to poor COVID-19 outcomes, also independently of age (23), it is known to be affected by sex, age, and comorbidities (24, 25). This may explain the stronger prognostic performance of CRP in our study, as our population was restricted to older patients with comorbidities. On the other hand, IP10 was linked to severe COVID-19 in early clinical reports of hospitalized patients (26), and has been described as a proxy of viral load for other viral infections, particularly HIV (27–29). While findings regarding the direct relationship between viral load and COVID-19 severity have been inconsistent, our results show higher viral loads in hospitalized patients, confirming viral load is a risk factor in this study population. Additionally, we observed a significant correlation between viral load and IP10 levels, indicating that IP10 may serve as a surrogate marker for disease severity related to viral load in our cohort.

CRP and IL6 levels are widely used in emergency departments in high-income settings as markers of inflammation and disease severity, primarily measured through laboratory assays like ELISA. Interestingly, when applying current reference cut-off values in our cohort, we obtained high sensitivity for both CRP and IL6 to predict hospitalization and severity, respectively. This aligns with the goal of achieving a high negative predictive value to ensure that no patient at risk is overlooked. Notably, novel point-of-care assays for rapid measurement of CRP, IL6, and IP10 are now available or in development, which can enable assessment of these biomarkers in primary health care and resource-limited settings. CRP, IL6, and IP10 could support clinical decision making, particularly the need of referral or admission, by identifying high-risk COVID-19 patients in an outpatient setting or emergency service. They could also inform the need of close follow-up of patients discharged home. Such risk stratification strategies could facilitate timely intervention and treatment, help reduce disease progression and risk of sequelae, while also optimizing resource allocation. To avoid unnecessary hospitalizations, cut-off values resulting in low specificity could be reviewed or combined with other risk factors to refine such risk stratification strategies.

Our results also suggest that some biomarkers, including suPAR, IL8, and IL1RA might have a better prognostic performance at identifying more severe COVID-19 in comparison to overall hospitalization, which included patients without oxygen requirement and no other truly severe manifestations. However, the frequency of more severe endpoints in our cohort was relatively low (only 5 patients required mechanical ventilation, 2 of whom died), limiting statistical power to draw strong conclusions. Indeed, we could not measure the association of biomarkers’ levels with the highest severity scores related to intubation and mortality, due to the low frequency of these events. In accordance, levels of sTREM1, which typically have been found to predict mortality in many life-threatening infections including COVID-19 (30–35), were not associated with hospitalization and severity in our study. Interestingly, lymphocyte levels in our study did not follow the expected trend with increasing severity, contrasting with most existing evidence (primarily from hospitalized patients), which identifies lymphopenia as a strong predictor of COVID-19 severity and mortality (36, 37). As lymphocyte levels typically decline over time as severity increases (38), lymphopenia may be a reliable late indicator of severe disease, but not an effective early predictor.

Of note, we examined biomarker kinetics from baseline to day 7 according to disease progression in a subset of participants with available biomarkers’ data at day 7, which has been largely unexplored. We observed a trend of increasing levels of ferritin and CRP in participants that required hospitalization within 28 days, regardless of their hospitalization status at day 7, compared to controls. Additionally, IL6 and IP10 showed a trend in faster normalization in participants discharged by day 7, compared to those still hospitalized. This secondary analysis was restricted to participants with available sample on day 7, in the context of challenging sample collection particularly in severe cases. While results may be impacted by the small sample size, these findings are relevant for monitoring disease progression and suggest that IL6 and IP10 may serve as timely indicators of recovery, warranting further investigation.

A main strength of our study is the use of a nested case-control design from a multicenter randomized clinical trial with consistent data collection and standardized outcome definitions and laboratory assays. Moreover, we compared a large panel of inflammatory, immune, and endothelial activation markers, including most of the key prognostic markers proposed in the literature, which allowed to draw strong conclusions on their early prognostic performance in mild-to-moderate COVID-19.

Our study has some limitations. First, the low incidence of mechanical ventilation and death in our study cohort limited our ability to assess the association between biomarkers and the most severe endpoints, including mortality risk. Second, the study was conducted during the first and second years of the COVID-19 pandemic and involved unvaccinated patients aged 50 or older, most of whom were immunocompetent. This limits the generalizability of our findings to the current population at highest risk of severe outcomes, mainly immunocompromised individuals with prior infections and full vaccination. Third, our nested case-control design did not involve matching to minimize confounding bias, given the highly homogeneous characteristics of the original cohort. Instead, we adjusted for relevant covariates in the statistical analysis. Finally, we did not evaluate the prognostic performance of biomarkers combinations or their integration with clinical severity indicators in risk prediction models.

In conclusion, higher levels of the biomarkers CRP, IL6, IP10, ferritin, IFNα, IL8, IL1RA, MCP1, and lower levels of RANTES measured within 7 days of symptoms onset were associated with increased risk of hospitalization due to COVID-19 progression. CRP and IL6 were the biomarkers with the highest discriminatory capacity for hospitalization, and severe COVID-19, respectively. These findings could guide management decisions in the context of new COVID-19 outbreaks to help guide triage and management decisions, including admission versus discharge and prioritization of early treatment, especially in those patients with risk factors or unvaccinated.

## Data Availability

The raw data supporting the conclusions of this article will be made available by the authors, without undue reservation.
