# Supplementary material for: Prognostic performance of early immune and endothelial activation markers in mild-to-moderate COVID-19 outpatients: a nested case-control study
Source: Front Immunol. 2024 Nov 27;15:1501872. doi: 10.3389/fimmu.2024.1501872 (PMC11631913; doi:10.3389/fimmu.2024.1501872)
Supplement: Supplementary file 1 [file DataSheet1.pdf]

Supplementary appendix to Prognostic performance of early immune and endothelial activation markers in mild-to-moderate COVID-19 outpatients: a nested case-control study

Table of contents

Table of contents 1
COnV-ert BMK Group of Authors 2
Figure S1. Study flowchart........ 3
Table S1. Performance metrics associated with different CRP and IL6 cut-off levels 4
Figure S2. Correlation between viral load and selected biomarkers at baseline 5
Table S2. Comparison of the mean change in levels of each biomarker from baseline to day 7 6
Table S3. Treatment effect on biomarker levels from baseline to day 7........ 7

COnV-ert BMK Group of Authors

Fight Infectious Diseases Foundation, Badalona, Spain

Gèlia Costes, MD; Mar Capdevila-Jáuregui, MD; Pamela Torrano-Soler, RN; Alba San José, RN; Bonaventura Clotet, PhD, Prof; Maria Ubals, PhD; Camila González-Beiras, PhD; Clara Suñer, PhD.

Hospital Universitari Germans Trias i Pujol, Badalona, Spain

Glòria Bonet Papell, MD

Emergency Department, Bellvitge University Hospital, Hospital de Llobregat, Spain

Pierre Malchair, MD; Aurema Otero, MD; Jose Carlos Ruibal Suarez, MD; Alvaro Zarauza Pellejero, MD; Ferran Llopis Roca, PhD; Orlando Rodriguez Cortez, MD; Vanesa Garcia Garcia, RN

Unitat de Suport a la Recerca de la Catalunya Central, Fundació Institut Universitari per a la recerca a l'Atenció Primària de Salut Jordi Gol i Gurina, Sant Fruitós de Bages, Spain

Anna Ruiz-Comellas, PhD; Anna Ramírez-Morros, MPH; Josep Vidal-Alaball, PhD

Salut Catalunya Central, Hospital de Berga, Berga, Spain

Joana Rodríguez Codina, RN; Rosa Amado Simon, MD

Barcelona Institute for Global Health (ISGlobal), Barcelona, Spain

Cristina Garcia-Mauriño, MD, PhD

Figure S1. Study flowchart.

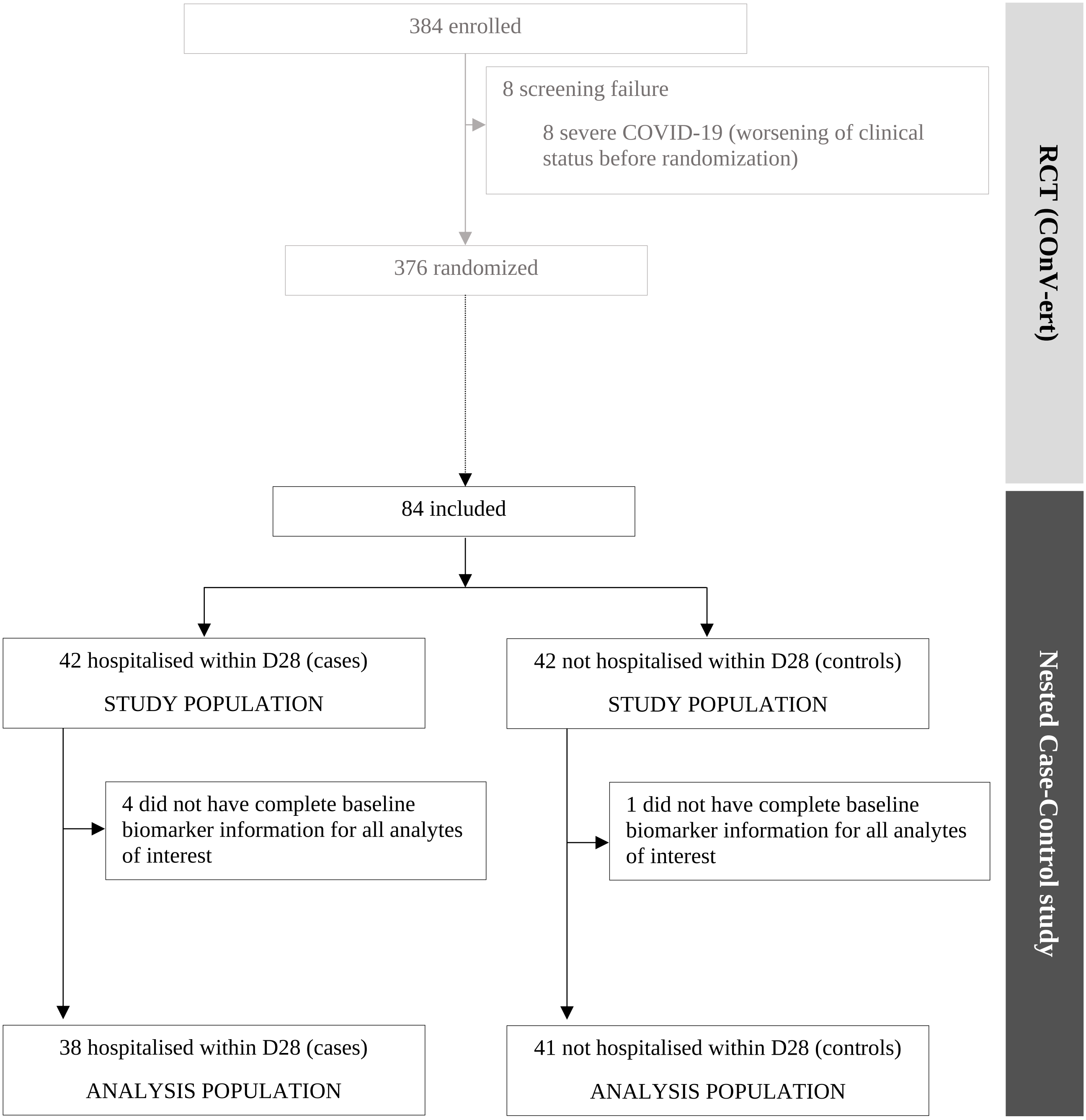

Table S1. Performance metrics associated with different CRP and IL6 cut-off levels

Biomarker	Outcome	Cut-off	Sensitivity (95% CI)	Specificity (95% CI)	LR+ (95% CI)	LR- (95% CI)	PPV (95% CI)	NPV (95% CI)
CRP	Hospitalization	≥5.0 mg/L*	89.5 (75.2, 97.1)	63.4 (46.9, 77.9)	2.45 (1.61, 3.71)	0.17 (0.06, 0.43)	69.4 (54.6, 81.7)	86.7 (69.3, 96.2)
IL6	Severe COVID-19	≥5.6.4 pg/mL*	92.3 (64.0, 99.8)	43.9 (31.7, 56.7)	1.65 (1.26, 2.15)	0.18 (0.03, 1.17)	24.5 (13.3, 38.9)	96.7 (82.8, 99.9)
CRP	Hospitalization	≥10.23 mg/L[¥]	76.3 (59.8, 88.6)	82.9 (67.9, 92.8)	4.47 (2.23, 8.98)	0.29 (0.16, 0.5)	80.6 (64.0, 91.8)	79.1 (64.0, 90.0)
IL6	Severe COVID-19	≥11.00 pg/mL[¥]	84.6 (54.6, 98.1)	66.7 (54.0, 77.8)	2.54 (1.68, 3.83)	0.23 (0.06, 0.84)	33.3 (18.0, 51.8)	95.7 (85.2, 99.5)

Legend: Different cut-off levels of CRP for hospitalization and IL6 for severe COVID-19, with their sensitivity, specificity, likelihood ratios (positive and negative), and predictive values (positive and negative).

*Laboratory reference ranges

[¥]Youden's index method

Figure S2. Correlation between viral load and selected biomarkers at baseline

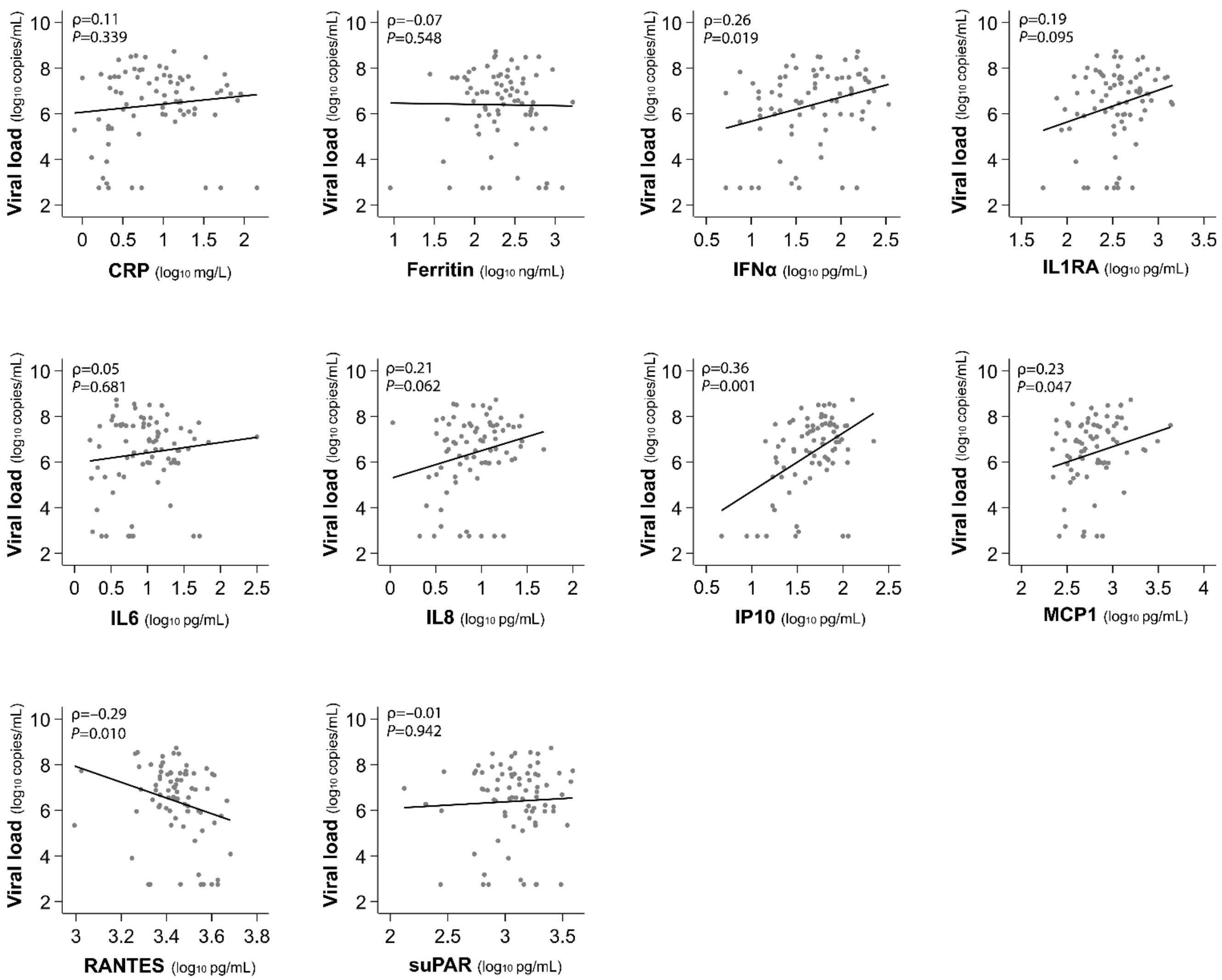

Legend: Correlation of baseline viral load (log_{10} copies/ml) and selected biomarkers (log_{10}).

Spearman's rank correlation coefficients, ρ (rho), and corresponding *p*-values are indicated in the figure.

Table S2. Comparison of the mean change in levels of each biomarker from baseline to day 7

	Non-hospitalized (controls)		Hospitalized (cases)					
	Non-hospitalized during the entire follow-up		Hospitalized but already discharged at day 7 (cases with shorter hospitalization)			Still hospitalized at day 7 (cases with longer hospitalization)		
	N	Difference D0-D7, mean (SD)	N	Difference D0-D7, mean (SD)	p-value	N	Difference D0-D7, mean (SD)	p-value
CRP	41	-0.05 (0.46)	12	0.28 (0.80)	0.073	10	0.19 (0.66)	0.225
Ferritin	33	0.06 (0.16)	12	0.24 (0.37)	0.093	10	0.29 (0.60)	0.038
IFNα	39	-0.09 (0.37)	6	-0.11 (0.30)	0.942	6	-0.54 (0.90)	0.019
IL6	41	-0.16 (0.36)	14	-0.18 (0.78)	0.908	9	0.11 (0.73)	0.162
IL8	39	-0.17 (0.30)	6	-0.53 (0.58)	0.016	6	-0.22 (0.41)	0.755
IL1RA	39	-0.07 (0.15)	6	-0.33 (0.57)	0.046	6	-0.06 (0.66)	0.922
IP10	39	-0.44 (0.27)	6	-0.61 (0.68)	0.228	6	-0.17 (0.32)	0.070
MCP1	39	0.01 (0.20)	6	-0.24 (0.34)	0.022	6	-0.16 (0.42)	0.107
RANTES	39	<0.01 (0.16)	6	0.08 (0.10)	0.238	6	-0.01 (0.10)	0.784
suPAR	39	-0.07 (0.14)	6	-0.10 (0.27)	0.757	6	-0.03 (0.41)	0.640

Legend: Biomarkers were $\log_{10}$ transformed, with concentrations expressed in pg/mL except for CRP (mg/L) and ferritin (ng/mL). Comparison of the mean difference of each biomarker level from baseline to day 7 between groups was estimated trough fitting linear mixed-effects models, using the individual as random effects in the intercept to adjust for intra-individual correlation and modeling time-group interaction effects.

Table S3. Treatment effect on biomarker levels from baseline to day 7

	Placebo		CCP		p-value
	N	Difference D0-D7, mean (SD)	N	Difference D0-D7, mean (SD)	
Angpt2	23	0.03 (0.07)	28	0.01 (0.10)	0.619
CRP	28	0.12 (0.61)	35	-0.01 (0.55)	0.368
D-dimer	28	-0.01 (0.35)	34	0.10 (0.22)	0.142
EGF	23	0.08 (0.28)	28	0.07 (0.32)	0.947
EOTAXIN	23	0.06 (0.27)	28	<0.01 (0.22)	0.344
Ferritin	24	0.19 (0.27)	31	0.10 (0.37)	0.280
FGF	23	-0.02 (0.22)	28	0.01 (0.30)	0.628
G-CSF	23	0.05 (0.39)	28	-0.03 (0.30)	0.406
GMCSF	23	0.18 (0.43)	28	0.02 (0.45)	0.191
HGF	23	0.07 (0.26)	28	0.10 (0.27)	0.654
IFNα	23	-0.06 (0.40)	28	-0.22 (0.50)	0.201
IFNγ	23	-0.14 (0.66)	28	-0.24 (0.76)	0.614
IL10	23	-0.29 (1.14)	28	0.03 (0.41)	0.165
IL12	23	-0.08 (0.16)	28	-0.24 (0.31)	0.029
IL13	23	-0.07 (0.37)	28	-0.03 (0.28)	0.697
IL17	23	0.05 (0.78)	28	0.21 (0.79)	0.444
IL1B	23	0.01 (0.35)	28	-0.01 (0.25)	0.771
IL1RA	23	<0.01 (0.27)	28	-0.17 (0.34)	0.046
IL2	23	0.18 (0.43)	28	0.07 (0.52)	0.427
IL2R	23	0.04 (0.26)	28	0.07 (0.23)	0.705
IL4	23	0.11 (0.51)	28	0.04 (0.55)	0.672
IL5	23	0.21 (0.43)	28	0.05 (0.35)	0.156
IL6 hospital	29	-0.02 (0.49)	35	-0.21 (0.56)	0.159
IL6 30-plex	23	0.02 (0.38)	28	-0.18 (0.62)	0.181
IL7	23	0.14 (0.25)	28	0.07 (0.20)	0.218
IL8 5-plex	23	-0.20 (0.45)	28	-0.24 (0.28)	0.718
IL8 30-plex	23	-0.16 (0.39)	28	-0.24 (0.32)	0.402
IP10	23	-0.35 (0.39)	28	-0.49 (0.31)	0.142
Lymphocytes	28	0.10 (0.16)	34	0.10 (0.20)	0.879
MCP1	23	0.01 (0.27)	28	-0.08 (0.26)	0.216
MIG	23	0.10 (0.31)	28	0.04 (0.19)	0.359
MIP1A	23	0.01 (0.43)	28	0.09 (0.29)	0.445
MIP1B	23	0.03 (0.26)	28	0.14 (0.33)	0.214
PCT	23	-0.07 (0.11)	28	-0.07 (0.15)	0.995
Prealbumin	29	-0.02 (0.20)	36	-0.01 (0.22)	0.741
RANTES	23	0.03 (0.16)	28	-0.01 (0.14)	0.328
sTREM1	23	0.08 (0.10)	28	0.09 (0.10)	0.692
suPAR	23	-0.04 (0.16)	28	-0.10 (0.23)	0.228
TNF	23	0.11 (0.56)	28	0.08 (0.53)	0.838
VEGF	23	-0.07 (0.40)	28	-0.06 (0.54)	0.918

Legend: Biomarkers were log_{10} transformed, with concentrations expressed in pg/mL except for CRP (mg/L), D-dimer (ng/mL), ferritin (ng/mL), lymphocytes ($x10^9$ cells/L), and prealbumin (mg/dL). Comparison of the mean difference of each biomarker level from baseline to day 7 between groups was estimated trough fitting linear mixed-effects models, using the individual as random effects in the intercept to adjust for intra-individual correlation and modeling time-group interaction effects.
